# A critical appraisal of the safety of bedinvetmab (Beransa), a canine antinerve growth factor monoclonal antibody

**DOI:** 10.1111/avj.70088

**Published:** 2026-05-05

**Authors:** X Yang, P Macarthur

**Affiliations:** ^1^ School of Veterinary Science University of Sydney Sydney New South Wales Australia

**Keywords:** bedinvetmab, canine, degenerative joint disease, nerve growth factor, osteoarthritis

## Abstract

**Clinical scenario:**

Canine osteoarthritis (OA) is a degenerative joint disease and is one of the most common chronic conditions in dogs and other species. The management of OA remains a longstanding focus in veterinary medicine. Traditionally, nonsteroidal anti‐inflammatory drugs (NSAIDs) have been the first‐line treatment option for canine OA. Recently, bedinvetmab, a canine‐specific monoclonal antibody (mAb) targeting the nerve growth factor (NGF), has been added as a pain management option for canine OA.

**Clinical question:**

Is monthly bedinvetmab injection safe in dogs over 12 months old with OA compared with other interventions?

**Summary of key findings:**

Four articles met the inclusion criteria of a structured literature search and investigated adverse events (AEs) associated with bedinvetmab. Two studies investigated a 3‐month treatment period and did not find an increase in AEs associated with bedinvetmab in comparison with the placebo control group. The third study compared the safety of bedinvetmab to meloxicam in a 2‐month treatment period, where bedinvetmab was found to have fewer associated AEs. The fourth study conducted a disproportionality analysis, and the musculoskeletal AEs were reported significantly more frequently in bedinvetmab‐treated dogs than other traditional therapeutics.

**Summary of appraisal:**

There is conflicting evidence regarding the safety of bedinvetmab, though stronger evidence supported the safety of bedinvetmab when administered for under 3 months.

**Future research:**

Future research should investigate the long‐term safety of bedinvetmab and incorporate radiographic imaging pre‐ and post‐treatment to monitor unusual joint changes.

**Abbreviations**
AE: adverse event
COI: canine orthopaedic index
CAT: critically appraised topic
CEBM: Centre for evidence‐based medicine
CBPI: canine brief pain inventory
COX: cyclooxygenase
DA: disproportionality analysis
mAb: monoclonal antibody
MSAERs: musculoskeletal adverse event reports
NGF: nerve growth factor
NSAIDs: nonsteroidal anti‐inflammatory drugs
OA: osteoarthritis
PIS: pain intereference score
PSS: pain severity score
RRR: proportional reporting ratio
RCT: randomized control trial
SC: subcutaneous
TrkA: tyrosine kinase receptor
VeDDRA: veterinary dictionary for drug related affairs

## Clinical scenario

Canine osteoarthritis (OA) is a chronic degenerative joint disease that commonly affects dogs, leading to pain, reduced mobility and a diminished quality of life. The condition is often diagnosed based on radiographic changes in the later stages of the disease, and early detection prior to the onset of clinical signs remains challenging.^1^ The estimated prevalence of canine OA varies depending on the study. Anderson et al^2^ reported a prevalence of 2.5%, whereas O'Neil et al^3^ claimed a rate of 6.6% among dogs seen in primary care practices in the United Kingdom. However, another study done in the United States found that 39.8% of the general young dog population – aged between 8 months to 4 years – showed osteoarthritic changes on screening radiographs, and 16.3% exhibited clinical joint pain. The true prevalence of canine OA, therefore, is likely underreported in primary care settings due to underdiagnosis and discrepancies in medical record‐keeping.^3^, ^4^ As the Australian pet dog population is estimated at 6.4 million, a substantial number of dogs may be suffering from OA.^5^ The growing awareness of chronic pain motivates owners to seek interventions that enhance their dog's mobility and reduce suffering.^6^

The management of OA remains a longstanding focus in veterinary medicine, with treatment strategies typically involving a multimodal approach such as physiotherapy, weight control program and nutritional supplement.^7^ Pharmacological interventions primarily target pain management, as chronic pain leads to central sensitization and emotional distress, perpetuating a vicious cycle that significantly impacts animal welfare.^8^ Traditionally, nonsteroidal anti‐inflammatory drugs (NSAIDs) have been the first‐line pharmacological option for canine OA. Their analgesic effect is achieved via inhibition of the enzyme cyclooxygenase (COX), which reduces production of prostaglandins that are involved in pain perception and sensitization. However, pain management is a constant trade‐off between safety and efficacy.^9^ NSAIDs have many side effects due to their inherent mechanism of action. Meloxicam, a commonly used NSAID licensed in the 1990s to manage OA, has a reported gastrointestinal adverse event rate of 12%^10^ to 15%.^9^ With few exceptions – such as Mavacoxib, which offers monthly dosing^11^ – most NSAIDs also require daily oral administration, which poses compliance issues for owners. This highlights that canine OA is a difficult problem, where no single treatment is appropriate for every dog. Therefore, there is a need for an alternative reliable therapeutic treatment to manage OA pain in canine patients whose NSAID treatment has failed for various reasons.

Since 2013, a canine‐specific monoclonal antibody (mAb) targeting the nerve growth factor (NGF) has emerged as a promising way for managing arthritic pain in dogs.^12^ NGF is a soluble signaling protein that plays an important role in nociception by binding to NGF‐specific tyrosine kinase receptor (TrkA).^13^ When NGF binds to TrkA expressed on the peripheral terminals of sensory nerve fibres, the NGF/TrkA complex is retrogradely transported to the cell body of sensory neurons. This triggers a variety of receptors and ion channels involved in nociception such as transient receptor potential vanilloid 1, acid‐sensing ion channels, bradykinin receptors and mechanotransducers, causing peripheral sensitization of afferent neurofibres.^13^ The NGF/TrkA complex also increases expression of pronociceptive neurotransmitters such as substance P, calcitonin gene‐related peptide and brain‐derived neurotrophic factor.^13^ In the periphery, NGF activates mast cells to release inflammatory mediators such as histamine, further sensitizing the afferent neurons.^14^ Studies have found that the NGF concentrations in synovial fluids of dogs with chronic lameness are significantly elevated compared to healthy dogs.^15^ Given the significant role of NGF in pronociceptive processes, neutralizing free NGF within the joints with anti‐NGF mAb can disrupt this pronociception signaling pathway and provide analgesic effect. In 2020, bedinvetmab (brand names Librela in United States and Beransa in Australia) became the first canine‐specific anti‐NGF mAb that was licensed in various countries for pain management in canine OA.

According to Zoetis Global Pharmacovigilance database (Feb 2021‐Jun 2024), over 18 million doses of bedinvetmab were sold globally.^16^ However, despite the popularity, the launch of the product has received much negative press coverage. The main reason being that the human equivalent of anti‐NGF mAb, tanezumab, has been found to increase the incidence of rapidly progressive osteoarthritis (RPOA), a rare but painful condition that is characterised by rapid bone destruction and loss of joint space.^17^ While the FDA halted the human clinical trials in 2012, so far, RPOA is not recognised in the Veterinary Dictionary for Drug Related Affairs (VeDDRA) coding. In Dec 2024, the FDA issued an open letter to veterinarians, alerting them to the 18 distinct safety signals identified in dogs using pharmacovigilance data collected postmarketing.^18^ To this day, the concerns relating to the safety of the drug remain highly debatable. Various studies have given conflicting evidence regarding the safety of bedinvetmab.^19^, ^20^, ^21^, ^22^

### Focused clinical question

Is monthly bedinvetmab injection safe in dogs over 12 months old with OA compared with alternative interventions?

## Materials and methods

### Terms used to guide search strategy

1. Patient: dogs over 12 months old diagnosed with OA
2. Intervention: monthly subcutaneous injection of bedinvetmab
3. Comparison: receiving an intervention other than bedinvetmab
4. Outcome: Adverse events

### Search terms identified for the PICO question

**Table:** 

Patient		Intervention	Comparator	Outcome
Species	Condition			
Dog Dogs Canine Canines Canis	Osteoarthritis OA Osteoarthritic Arthritis Arthritic Degenerative joint disease DJD	Bedinvetmab Librela Beransa Nerve growth factor NGF Monoclonal antibody	Other intervention	Adverse events

The primary data were obtained using the search strategies listed in Table 1. The databases included PubMed and Web of Science to include all potentially relevant studies. After the first round of screening, any duplicate studies were removed. Subsequently, a set of inclusion and exclusion criteria was created based on the PICO question (Table 2). The second round of screening removed any studies irrelevant to the PICO‐based criteria (only on a title and abstract basis), and the remaining reports were retrieved for full‐text evaluation.

**Table 1:** Databases used, literature dates covered and search terms

Databases searched and dates covered	PubMed (2015–2025) Web of Science (2015–2025)
Search terms for each database	PubMed: (canine OR dog OR Canis OR canines OR dogs) AND (osteoarthritis OR OA OR osteoarthritic OR arthritis OR arthritic OR degenerative joint disease OR DJD) AND (bedinvetmab OR Librela OR Beransa OR monoclonal antibody OR nerve growth factor OR NGF) = 39 results Web of Science: (canine OR dog OR Canis OR canines OR dogs) AND (osteoarthritis OR OA OR osteoarthritic OR arthritis OR arthritic OR degenerative joint disease OR DJD) AND (bedinvetmab OR Librela OR Beransa OR monoclonal antibody OR nerve growth factor OR NGF) = 43 results

**Table 2:** Inclusion and exclusion criteria used to eliminate literature not relevant to the PICO question

Inclusion	Studies with patients diagnosed with osteoarthritis Patients over 12 months of age Studies that recorded adverse events associated with bedinvetmab administration Studies with monthly bedinvetmab injection as the intervention Literature written in English
Exclusion	Studies that did not use monthly injection of bedinvetmab as the intervention Studies that recruited healthy patients Studies with patients younger than 12 months of age Literature not written in English

## Results

The initial database search yielded 39 papers from PubMed and 49 papers from Web of Science. The removal of duplicate studies reduced the number of papers to a total of 60. The second round of screening based on title and abstract further removed 55 papers, leaving 5 remaining papers for full‐text evaluation (Table 3).

**Table 3:** Summary of study designs of articles retrieved for full‐text evaluation

Level of evidence	Study design	Study
1b	RCT	Corral et al^19^
1b	RCT	Michels et al^20^
1b	RCT	Innes et al^21^
1b	RCT	Krautmann et al^23^
4	DA	Farrell et al^22^

The level of evidence was determined by using the Levels of Evidence for Therapeutic Studies, set by the Centre for Evidence‐Based Medicine (CEBM).^24^ Krautmann et al^23^ was removed for recruiting healthy participants instead of patients with existing OA. DA, disproportionality analysis; RCT, randomised control trial.

After further inspection, Krautmann et al^23^ was removed because the study recruited healthy patients instead of patients diagnosed with OA, based on exclusion criteria of the PICO question. Studies done by Corral et al, Michels et al, Innes et al and Farrell et al were used for the purpose of this critically appraised topic (CAT) due to adverse events being recorded to analyse the safety of bedinvetmab.^19^, ^20^, ^21^, ^22^ The summary of evidence from each of the included studies is listed in Table 4.

**Table 4:** Summary of evidence

Author	Corral et al^19^	Michels et al^20^	Innes et al^21^	Farrell et al^22^
Year	2021	2023	2025	2025
Design	RCT	RCT	RCT	DA
Population	A total of 287 dogs (165 pure‐bred and 122 mixed breeds; 133 males and 154 females; 61% desexed; age = 8.9 (1.0–17.5) years, weight = 26.7 (1.7–66.0) kg, baseline CBPI PIS score = 5.39 [0.11], baseline CBPI PSS score = 4.75 [0.10]) were used in this study. All dogs had clinical and radiographic evidence of OA and had no other uncontrolled concurrent disease that could confound the evaluation of bedinvetmab safety and efficacy.	A total of 272 dogs (171 pure‐breds and 101 mixed breeds; 117 males and 155 females; 95.6% desexed; age = 9.5 [2.9] years, weight = 28.3 [13.6] kg, baseline CBPI PIS score = 5.97 [1.86], baseline CBPI PSS score = 5.24 [1.66]) were used in this study. All dogs had clinical and radiographic evidence of OA at a screening visit and had no uncontrolled concurrent disease that could confound the evaluation of bedinvetmab safety and efficacy.	A total of 101 dogs (21 crossbreeds, 27 labrador, 53 other; 51 males and 50 females; 82% desexed; age = 10.5 [2.6] years, weight = 23.9 [9.5] kg, body condition score = 5.6 [1.1], pretreatment COI = 40.0 [9.4]) were used in this study. All dogs had clinical and radiographic evidence of OA during a screening visit and an owner‐assessed COI score 26 or higher.	A total of 878 MSAERs (789 attributed to Librela and 89 attributed to Rimadyl, Metacam, Previcox, Onsior, Galliprant or Daxocox) were identified between 20 May 2021 and 31 December 2024 in the European Medicines Agency's EudraVigilance database. These reports did not entail any coadministration of Librela or confounding neurological and/or systemic/neoplastic diagnoses.
Intervention investigated	During the comparative phase, eligible dogs were randomly allocated to placebo group (n = 146) or bedinvetmab group (n = 141). The placebo group received monthly SC injection of saline whereas the bedinvetmab group received monthly SC injection of bedinvetmab (0.5–1.0 mg/kg; Librela; Zoetis Inc., MI, USA) for 3 months. Afterwards, bedinvetmab‐treated dogs that responded positively based on owner and veterinarian assessments (n = 89) proceeded to the open‐label continuation phase, during which they were administered up to six additional doses of bedinvetmab.	Eligible dogs were randomly allocated to placebo group (n = 137) or bedinvetmab group (n = 135). The placebo group received SC injection of saline, whereas the bedinvetmab group received monthly SC injection of bedinvetmab (0.5–1.0 mg/kg; Librela; Zoetis Inc., MI, USA) for 3 months.	Eligible dogs were randomly allocated to the bedinvetmab group (n = 52) or the meloxicam group (n = 48). The bedinvetmab group received monthly SC bedinvetmab injection (0.5–1.0 mg/kg; Librela; Zoetis Inc., MI, USA) administered by a veterinarian for 2 months. The meloxicam group received an initial SC injection of meloxicam (0.2 mg/kg) administered by a veterinarian, then received daily oral meloxicam suspension (0.1 mg/kg) administered by the owner for the remainder of the study.	The reports were used to conduct a MSAER disproportionality analysis.
Outcome measures	Frequencies of dogs with at least one AE were summarised by clinical sign. The AEs were clustered in organ classes following the VeDDRA cording.	Frequencies of dogs with at least one AE were summarised by clinical sign. The AEs were clustered in organ classes following the VeDDRA cording.	Frequencies of dogs with AEs were summarissed by preferred term and system organ class clinical sign following the VeDDRA coding.	The accumulation of MSAERs (ligament/tendon injury, polyarthritis, fracture, musculoskeletal neoplasia and septic arthritis) for Librela and comparator drugs were demonstrated in a timeline fashion.
Main findings	41 out of 146 dogs (28.1%) in the placebo group, 26 out of 141 dogs (18.4%) in the bedinvetmab group and 23 out of 89 dogs (25.8%) in the continuation group experienced one or more AEs. AEs were categoried into 11 organ classes according to VeDDRA and abnormal laboratory results. The frequency of AEs within each category occurred at similar rate in all three groups across all organ classes.	86 out of 137 dogs (62.8) in the placebo group and 79 out of 135 dogs (58.5) in the bedinvetmab group experienced one or more AEs. AEs were categorised into 11 organ classes according to VeDDRA. The frequency of AE within each class occurred at a similar rate between groups and not considered treatment related.	17 out of 49 dogs (34.7%) in the meloxicam group and 4 out of 52 dogs (7.7%) in the bedinvetmab group experienced one or more AEs. The organ class of AEs associated with meloxicam group included skin and appendages disorders (n = 1), digestive tract disorders (n = 8), systemic disorders (n = 1), musculoskeletal disorders (n = 3), behavioural disorders (n = 1) and neurological disorders (n = 3). The organ class of AEs associated with bedinvetmab group included skin and appendage disorders (n = 1), systemic disorders (n = 1) and musculoskeletal disorders (n = 2). The number needed to harm for meloxicam compared to bedinvetmab was 5 (95% confidence interval 2.8–27.2).	MSAERs attributed to Librela was ~9 times more frequently reported than the combined total of MSAERs attributed to the six comparator drugs.
Level of evidence	1b	1b	1b	4
Limitations	During the continuation phase, 10 dogs were withdrawn due to the development of unrelated medical conditions. One case developed neurological pelvic limb paresis that led to euthanasia. Two cases developed coronoid process fracture and distal humeral condylar fracture respectively. These are all neurological or musculoskeletal conditions that should be labeled as AEs instead of being dismissed as unrelated health events. There was a clear upward trend in the group of systemic disorders (1.4% in placebo group, 5.0% in the bedinvetmab comparative group, and 11.2% in the bedinvetmab continuation group). The study dismissed this as an incidental finding without any explanation.	No clear limitation was identified for this study.	The compliance for the two comparative groups was unmatched. While the compliance for bedinvetmab group was good because the injection was administered by vets during visits, the compliance for meloxicam group was challenging to assess because the daily tablets were administered by owners themselves. The compliance issue may have impacted the study result. The study methodology lacked blinding to both participants and outcome assessors, which is a confounding bias that could have impacted study outcome. The investigators have already hypothesised bedinvetmab to be safer than meloxicam prior to study commencement, which could have introduced ascertainment bias when conducting the study.	The study produced lower‐level evidence of outcome because it had no control, no blinding and no randomisation. It was not adjusted for confounding and was subject to reporting bias. The study directly compared the number of reports instead of the incidence rate of MSAEs because it did not take into account the number of dogs who have received treatment. This methodology is inherently problematic because a popular drug used widely can have more reports than a drug used rarely, without necessarily being more dangerous. The methodology of filtering MSAEs from the database was not documented and was done by a single author.

The strengths and limitations sections of the table are a summarized finding of the CASP checklists completed for each appraised study, which can be found in Supporting Information S1–S4. AE, adverse events; CBPI, canine brief pain inventory; COI, canine orthopaedic index; DA, disproportionality analysis; MSAER, musculoskeletal adverse event report; OA, osteoarthritis; PIS, pain interference score (0–10); PSS, pain severity score (0–10); RCT, randomiszed control trial; SC, subcutaneous; VeDDRA, Veterinary Dictionary for Drug Related Affairs.

### Best evidence

The studies included were identified as the best match in accordance with our predetermined inclusion and exclusion criteria. These studies compared the safety of bedinvetmab with either a placebo or one or more interventions in OA patients. All studies used the number of adverse events as their outcome measures.

## Critical appraisal of evidence

The aim of this CAT was to determine the safety profile of bedinvetmab in OA patients and any associated adverse health effects. Out of the 4 studies^19^, ^20^, ^21^, ^22^ examined in this CAT, the 3 RCT studies^19^, ^20^, ^21^ supported the notion that bedinvetmab was safe to administer in dogs. Specifically, two studies^19^, ^20^ reported that bedinvetmab did not cause a rise in observed AEs compared to the placebo control group, and one study^21^ reported bedinvetmab to cause less gastrointestinal, behavioral and neurological AEs compared to meloxicam. However, the last study^22^ out of the 4 disagreed with the conclusion of the other three. The DA done using pharmacovigilance data demonstrated that musculoskeletal AEs associated with bedinvetmab were reported ~9 times more frequently than six comparator drugs combined, suggesting that the new drug was unsafe compared to traditional NSAIDs.^22^

The duration of intervention differed across the studies. Farrell et al^22^ processed the musculoskeletal adverse event reports (MSAERs) submitted from 20 May 2021 to 31 December 2024, which was over three and a half years. However, the reports did not reflect the length of treatment each patient has undergone before an AE was recognied. Innes et al^21^ used a 2‐month intervention period, with monthly administrations of bedinvetmab for the bedinvetmab group and daily meloxicam suspension for the comparison group. Both Corral et al^19^ and Michels et al^20^ had a placebo‐controlled intervention period of 3 months, with monthly subcutaneous (SC) injections of bedinvetmab or saline. Corral et al^19^ had a continuation phase in which patients in the bedinvetmab group received up to 6 additional doses of bedinvetmab. The combined 9‐month period is by far the longest intervention period available in published studies. As bedinvetmab is indicated for the management of a chronic condition like OA, the long‐term safety profile of bedinvetmab is just as important as short term. Since most studies examined by this CAT only investigated the AEs of bedinvetmab for 3 months or less, available evidence only assessed the short‐term safety of bedinvetmab. While Corral et al^19^ reported there to be no significant increase in AEs during the 6‐month continuation phase, the result excluded 10 dogs out of the 89 due to the development of unrelated medical medication. However, three of the withdrawn cases could potentially be counted as AEs because NGF/TrkA signaling pathway may be involved in those conditions. One case involved euthanasia due to neurological pelvic limb paresis. Though not proven in dogs, the knockout of NGF in adult mice produced animals with skeletal muscle dystrophy,^25^ so neurological paresis should be marked as a risk factor for anti‐NGF suppression. The other two cases involved coronoid process fracture and distal humeral condyle fracture, respectively. A study has found that administration of exogenous NGF to mice significantly increases load‐induced bone formation, whereas eliminating the TrkA signaling greatly reduces load‐induced bone formation.^26^ Neutralization of NGF long term can impair the normal adaptive response of bone to mechanical loading and therefore potentially render certain bones more susceptible to fracture. In addition, NGF has been found to participate in angiogenesis^27^ and subsequent callus formation in rats.^28^ Specifically, NGF/TrkA is expressed at the chondro‐osseous transition zone during endogenous endochondral fracture repair, and exogenous injection of beta‐NGF accelerates fracture repair by promoting cartilage to bone conversion.^29^ Consequently, suppression of the TrkA signaling may prolong bone healing. Due to the important role of NGF in bone metabolism, one cannot confidently exclude the possibility that long‐term suppression of NGF by bedinvetmab has contributed to the fracture in those two cases. Given the differences among these 4 studies,^19^, ^20^, ^21^, ^22^ one can only be assured of safe bedinvetmab use for 3 months or under in OA dogs. The long‐term safety profile of bedinvetmab remains largely undetermined.

All 4 studies^19^, ^20^, ^21^, ^22^ evaluated the safety of bedinvetmab via the number of AE occurrences. While three studies^19^, ^20^, ^21^ categorized AEs according to organ classes defined by VeDDRA coding, Farrell et al^22^ focused only on the musculoskeletal AEs. Counting the number of AEs is an acceptable evaluation of safety in the RCTs because the number of test subjects was known, and therefore, the incidence rate could be calculated and compared. However, the same design is inherently problematic for a DA like Farrell et al because the number of patients exposed to the drug and the duration of exposure were not incorporated into the analysis. Lacking a denominator, DA could not be used to reflect the true incidence rate of AEs, as a safe drug used extensively can have more reports than a dangerous one taken rarely. Instead, the proportional reporting ratio (RRR), the proportion of a specific AE out of all reported AEs for a drug, may have been a more objective comparator to evaluate the safety profile for drugs of interest.^30^ In addition to analyzing AE occurrences, Farrell et al^22^ also assessed the severity and outcome data for each bedinvetmab associated MSAERs, which was not done in the RCTs.^19^, ^20^, ^21^ MSAERs such as stiffness and head down were mostly marked as not serious whereas fractures and polyarthritis were mostly marked as serious and resulted in death in about 20% of the reported cases.^22^ Since the number of AEs do not reflect their impact on animal welfare, veterinary clinicians should consider implementing additional outcome measures to assess the AEs of a new treatment, such as serious/insignificant, recovering/ongoing, sequelae caused by AEs, fatality and so on.

With regard to study design, Corral et al,^19^ Michels et al^20^ and Innes et al^21^ were all RCTs while Farrell et al^22^ conducted a DA. According to the level of evidence guide set by CEBM,^24^ RCTs provided level 1b evidence and DAs level 4 evidence. Therefore, the results generated by the three RCT studies are much more reliable than the DA, which is also the only study that generated conflicting results. DAs are inherently biased due to the lack of blinding.^31^ Beransa (Librela) has generated considerable debate among veterinary professionals and pet owners since its launch. Misinformation about canine RPOA is prevalent even though it is not defined in dogs and has no proven causation linked to bedinvetmab. The spread and impact of online misinformation can lead to panic and undermine pet owners' trust in veterinary professionals,^32^ causing them to reject the use of bedinvetmab in necessary situations. Such negative press coverage may have also led to increased AE reporting rates compared to traditional NSAIDs, artificially inflating signal detection in Farrell et al.^22^ Although it is impossible to completely remove the impact of social perception, veterinarians should actively debunk rumors, backing their clinical decisions with research‐based information.^32^ Besides the reporting bias, pharmacovigilance data also lack critical details on duration of drug exposure, variabilities in study population, and threshold definition, which can all contribute to a biased result. As Lloyd has pointed out in their^30^ commentary of Farrell et al, the filtering criteria of AEs was not specified in the paper and was conducted by a single author instead of multiple investigators. The absence of transparent criteria and lack of reviewers can introduce substantial selection bias that impacts interpreted results. The multiple biases present in the study design, plus the method of using the sum of AE occurrences instead of incidence rate as a measurement of safety, make the evidence generated by Farrell et al less reliable. As a result, despite strongly suggesting that bedinvetmab is unsafe compared to traditional NSAIDs, Farrell et al could not draw a definitive conclusion regarding the overall safety of bedinvetmab. Conversely, one should point out that the three RCTs^19^, ^20^, ^21^ were all fully funded by Zoetis, especially with Corral et al^19^ and Michels et al^20^ being the premarketing trials for European and America respectively. Such conflict of interest needs to be recognised and declared.

As mentioned previously, the most well‐known AE of anti‐NGF mAb in human patients is RPOA, which is also where major arguments with regard to the safety profile of bedinvetmab stem from. RPOA is a rare but serious AE that resulted in the FDA halting the clinical trials for tanezumab. RPOA can only be diagnosed by radiographic imaging. In human trials, RPOA occurred most frequently after long‐term use of anti‐NGF. The incidence rate increased dramatically during week 56 imaging compared to week 24 and continued to rise during the follow‐up period.^33^ Clinicians therefore identified the period between week 24 and week 56 to be of the highest risk for RPOA.^33^ The most widely accepted definition of RPOA as proposed by Lequesne describes it as the loss of joint space of 2 mm per year or loss of 50% of joint space in 1 year.^34^ However, RPOA is not currently recognised in dogs, as such definition is not directly applicable to canine patients due to the drastic difference in joint sizes. Additionally, the rapidly narrowing of joint space in human patients is characterised by limited or no osteophyte formation, which can progress to joint erosion and cartilage and bone lysis in as little as 6 months.^35^ In the case series of suspected canine RPOA reported by Farrell et al, 9 cases had radiograph/CT imaging of affected joints before and after bedinvetmab injection for comparison.^22^ The majority of patients (7 out of 9) showed joint changes that could be characterised as marked periosteal reaction or osteophytosis, while others experienced severe joint erosion.^22^ Iff et al also reported a case of suspected RPOA in a canine patient, in which CT and radiograph results showed massive proliferative as well as erosive changes.^35^ This discrepancy between human and canine patients suggests that the pattern of rapid joint degeneration observed in dogs may not conform to the characterization of RPOA described in humans, and therefore, the term RPOA should be used cautiously. However, it remains possible that a form of RPOA does occur in dogs but manifests with different radiographic characteristics compared to the human condition. Consequently, further research is needed to better define and group cases involving marked osteophytosis, periosteal changes and erosions in canine joints, allowing them to be reported under an appropriate diagnostic term within VeDDRA, even if distinct from human RPOA. Additionally, since erosive joint disease has been documented in dogs,^22^, ^36^ albeit less common than extensive bone proliferation, one cannot rule out the possibility that dogs exhibit a form of rapid joint destruction resembling human RPOA. A synovial fluid analysis should be performed in suspected cases to exclude alternative etiologies such as immune‐mediated polyarthritis and septic arthritis. Given the available reports of suspected RPOA in canine patients, future research should incorporate radiographic imaging pre‐ and postintervention and during the follow‐up period so that the unusual joint changes can be identified and reported.

In summary, according to existing evidence, one can relatively confidently conclude that monthly bedinvetmab injection is not associated with increased AEs when administered for under 3 months. It is also associated with a smaller number of AEs compared to meloxicam when administered for under 2 months. This critical appraisal agrees with previous literature that suggests bedinvetmab is not associated with increased AEs in patients when used short term. However, substantial gaps in knowledge remain, especially with regard to the long‐term safety profile of bedinvetmab and its causal relationship with rare but severe AEs. We suggest practicing clinicians weigh the benefits against potential risks case‐by‐case before treating their patients with bedinvetmab. While the short‐term safety of bedinvetmab is assured in Corral et al and Michels et al, canine OA is a chronic condition that requires long‐term surveillance of adverse effects. Future research should aim to examine the safety of bedinvetmab for 6 months or longer. Future research should also strive to detect, group and define unusual joint changes by incorporating radiographic imaging pre‐ and post‐treatment and during follow‐up period. High‐quality research such as RCTs and cohort studies are preferred over low‐quality research so that a reliable and comprehensive evaluation of the safety of bedinvetmab can be generated. This CAT should be reviewed in 3 years to determine whether additional research evidence has been published that could aid in answering the focused clinical question.

## Conflicts of interest and sources of funding

The authors declare no conflicts of interest or sources of funding for the work presented here.

## Supporting information


**Data S1.** Supporting Information.

## Data Availability

Data sharing not applicable to this article as no datasets were generated or analysed during the current study.
