# Supplementary material for: A critical appraisal of the safety of bedinvetmab (Beransa), a canine antinerve growth factor monoclonal antibody
Source: Aust Vet J. 2026 May 5;104(8):549–56. doi: 10.1111/avj.70088 (PMC13428660; doi:10.1111/avj.70088)
Supplement: Supplementary file 1 — Data S1. Supporting Information. [file AVJ-104-549-s001.docx]

**Supporting information**

**For**

**A critical appraisal of the safety of bedinvetmab (beransa), a canine anti-nerve growth factor monoclonal antibody**

**Caption:** This file contains four Critical Appraisal Skills Programme (CASP) checklists completed for each of the studies included in this critical appraised topic. The checklist for randomized controlled trials is used for Corral et al, Michels et al, and Innes et al respectively, whereas the checklist for case control studies is used for Farrell et al. These checklists document the methodological quality and risk of bias assessment for each article.

**Supporting Method S1.**

CASP Checklist:

For Randomised Controlled Trials (RCTs)

| **Reviewer Name:** | Xingyu Yang |
| --- | --- |
| **Paper Title:** | A prospective, randomized, blinded, placebo-controlled multisite clinical study of bedinvetmab, a canine monoclonal antibody targeting nerve growth factor, in dogs with osteoarthritis |
| **Author:** | Corral MJ, Moyaert H, Fernandes T, Escalada M, Kira S Tena J, Walters RR, et al |
| **Web Link:** | https://www.sciencedirect.com/science/article/pii/S146729872100204 |
| **Appraisal Date:** | 06/08/2025 |

| **Section A Is the basic study design valid for a randomised controlled trial?** | |
| --- | --- |
| 1. Did the study address a clearly formulated research question? | Yes  No  Can’t Tell |
| *CONSIDER:*  *Was the study designed to assess the outcomes of an intervention?*  *Is the research question ‘formulated’ in terms of:*   - *Population studied* - *Intervention given* - *Comparator chosen* - *Outcomes measured?* | |
| 1. Was the assignment of participants to interventions randomised? | Yes  No  Can’t Tell |
| *CONSIDER:*   - *How was randomisation carried out? Was the method appropriate?* - *Was randomisation sufficient to eliminate systematic bias?* - *Was the allocation sequence concealed from investigators and participants?* | |
| 1. Were all participants who entered the study accounted for at its conclusion? | Yes  No  Can’t Tell  **All animals, including withdrew cases, were included in the safety data analysis.** |
| *CONSIDER:*   - *Were losses to follow-up and exclusions after randomisation accounted for?* - *Were participants analysed in the study groups to which they were randomised (intention-to-treat analysis)?* - *Was the study stopped early? If so, what was the reason?* | |
| **Section B Was the study methodologically sound?** | |
| 1. (a) Were the participants ‘blind’ to intervention they were given? | Yes  No  Can’t Tell |
| (b) Were the investigators ‘blind’ to the intervention they were giving to participants? | Yes  No  Can’t Tell |
| (c) Were the people assessing/analysing outcome/s ‘blinded’? | Yes  No  Can’t Tell |
| 1. Were the study groups similar at the start of the randomised controlled trial? | Yes  No  Can’t Tell |
| *CONSIDER:*   - *Were the baseline characteristics of each study group (e.g. age, sex, socio-economic group) clearly set out?* - *Were there any differences between the study groups that could affect the outcome/s?* | |
| 1. Apart from the experimental intervention, did each study group receive the same level of care (that is, were they treated equally)? | Yes  No  Can’t Tell |
| *CONSIDER:*   - *Was there a clearly defined study protocol?* - *If any additional interventions were given (e.g. tests or treatments), were they similar between the study groups?* - *Were the follow-up intervals the same for each study group?* | |
| **Section C: What are the results?** | |
| 1. Were the effects of intervention reported comprehensively? | Yes  No  Can’t Tell   - **AEs were recorded during monthly follow-up interval for each group.** - **The frequency of AE for each group was clustered in organ classes following the VeDDRA coding and presented neatly in a table.** - **In open-label continuation phase, 10 dogs were withdrawn due to the development of unrelated medical conditions. One case developed neurological deficits due to thoracic vertebrae 11-12 spinal collapse, which should be marked as a risk factor for bedinvetmab administration. One case developed pelvic limb paresis that led to euthanasia. Two cases developed coronoid process fracture and distal humeral condylar fracture respectively. These are neurological and musculoskeletal conditions that should be labeled as AEs instead of being dismissed as unrelated health events.** - **The study concluded all AEs were not related to bedinvetmab administration without showing any statistical test. There was a clear upward trend in systemic disorders (1.4% in placebo group, 5.0% in bedinvetmab comparative group, and 11.2% in bedinvetmab continuation group). The study dismissed them as incidental finding without any explanation.** - **Potential bias: The study, being the European pre-marketing trial, may be tempted to disguise severe AEs or dismiss AEs as unimportant in order to push the Librela into market.** |
| *CONSIDER:*   - *Was a power calculation undertaken?* - *What outcomes were measured, and were they clearly specified?* - *How were the results expressed? For binary outcomes, were relative and absolute effects reported?* - *Were the results reported for each outcome in each study group at each follow-up interval?* - *Was there any missing or incomplete data?* - *Was there differential drop-out between the study groups that could affect the results?* - *Were potential sources of bias identified?* - *Which statistical tests were used?* - *Were p values reported?* | |
| 1. Was the precision of the estimate of the intervention or treatment effect reported? | Yes  No  Can’t Tell  **No statistical test was done for the safety assessment section of the study.** |
| *CONSIDER:*   - *Were confidence intervals (CIs) reported?* | |
| 1. Do the benefits of the experimental intervention outweigh the harms and costs? | Yes  No  Can’t Tell   - **21 dogs out of 146 in placebo group did not complete the comparative study due to worsening clinical signs of OA. Although placebo group served as a crucial comparison for treatment group, ~1 in 7 dogs in placebo group suffered from OA pain to the point of withdrawal.** - **11 dogs out of 89 in continuation phase did not complete the comparative study (1 due to worsening OA, 10 due to unrelated medical conditions though upon closer inspection some dismissals were questionable).** |
| *CONSIDER:*   - *What was the size of the intervention or treatment effect?* - *Were harms or unintended effects reported for each study group?* - *Was a cost-effectiveness analysis undertaken? (Cost-effectiveness analysis allows a comparison to be made between different interventions used in the care of the same condition or problem.)* | |
| **Section D: Will the results help locally?** | |
| 1. Can the results be applied to your local population/in your context? | Yes  No  Can’t Tell |
| *CONSIDER:*   - *Are the study participants similar to the people in your care?* - *Would any differences between your population and the study participants alter the outcomes reported in the study?* - *Are the outcomes important to your population?* - *Are there any outcomes you would have wanted information on that have not been studied or reported?* - *Are there any limitations of the study that would affect your decision?* | |
| 1. Would the experimental intervention provide greater value to the people in your care than any of the existing interventions? | Yes  No  Can’t Tell |
| *CONSIDER:*   - *What resources are needed to introduce this intervention taking into account time, finances, and skills development or training needs?* - *Are you able to disinvest resources in one or more existing interventions in order to be able to re-invest in the new intervention?* | |

| **APPRAISAL SUMMARY**: *List key points from your critical appraisal that need to be considered when assessing the validity of the results and their usefulness in decision-making.* | | |
| --- | --- | --- |
| **Positive/Methodologically sound** | **Negative/Relatively poor methodology** | **Unknowns** |
| - **This study is the only published study that investigated the safety of bedinvetmab in dogs for up to 9 months.** | - **In open-label continuation phase, 10 dogs were withdrawn due to the development of unrelated medical conditions. One case developed neurological deficits due to thoracic vertebrae 11-12 spinal collapse, which should be marked as a risk factor for bedinvetmab administration. One case developed pelvic limb paresis that led to euthanasia. Two cases developed coronoid process fracture and distal humeral condylar fracture respectively. These are neurological and musculoskeletal conditions that should be labeled as AEs instead of being dismissed as unrelated health events.** - **The study concluded all AEs were not related to bedinvetmab administration without showing any statistical test. There was a clear upward trend in systemic disorders (1.4% in placebo group, 5.0% in bedinvetmab comparative group, and 11.2% in bedinvetmab continuation group) and neurological disorders (0 in both placebo and bedinvetmab comparative group, and 4.5% in bedinvetmab continuation group). The study dismissed them as incidental finding without any explanation.** | - **The study had radiographic evidence of OA pre-treatment but did not conduct radiography post-termination of treatment. RPOA is rare adverse reaction described in human anti-NGF mAb, which is mainly diagnosed by radiographs. Without post-termination radiograph, the study cannot rule out the possibility of RPOA being a potential AE of bedinvetmab**. |

**Supporting Method S2.**

CASP Checklist:

For Randomised Controlled Trials (RCTs)

| **Reviewer Name:** | Xingyu Yang |
| --- | --- |
| **Paper Title:** | A prospective, randomized, double-blind, placebo-controlled multisite, parallel-group field study in dogs with osteoarthritis conducted in the United States of America evaluating bedinvetmab, a canine anti-nerve growth factor monoclonal antibody |
| **Author:** | Gina M. Michels, Nicole A. Honsberger, Rodney R. Walters, Jezaniah Kira S. Tena, Dawn M. Cleaver |
| **Web Link:** | <https://www.vaajournal.org/article/S1467-2987(23)00103-4/fulltext> |
| **Appraisal Date:** | 14/08/2025 |

| **Section A Is the basic study design valid for a randomised controlled trial?** | |
| --- | --- |
| 1. Did the study address a clearly formulated research question? | Yes  No  Can’t Tell |
| *CONSIDER:*  *Was the study designed to assess the outcomes of an intervention?*  *Is the research question ‘formulated’ in terms of:*   - *Population studied* - *Intervention given* - *Comparator chosen* - *Outcomes measured?* | |
| 1. Was the assignment of participants to interventions randomised? | Yes  No  Can’t Tell |
| *CONSIDER:*   - *How was randomisation carried out? Was the method appropriate?* - *Was randomisation sufficient to eliminate systematic bias?* - *Was the allocation sequence concealed from investigators and participants?* | |
| 1. Were all participants who entered the study accounted for at its conclusion? | Yes  No  Can’t Tell |
| *CONSIDER:*   - *Were losses to follow-up and exclusions after randomisation accounted for?* - *Were participants analysed in the study groups to which they were randomised (intention-to-treat analysis)?* - *Was the study stopped early? If so, what was the reason?* | |
| **Section B Was the study methodologically sound?** | |
| 1. (a) Were the participants ‘blind’ to intervention they were given? | Yes  No  Can’t Tell |
| (b) Were the investigators ‘blind’ to the intervention they were giving to participants? | Yes  No  Can’t Tell |
| (c) Were the people assessing/analysing outcome/s ‘blinded’? | Yes  No  Can’t Tell |
| 1. Were the study groups similar at the start of the randomised controlled trial? | Yes  No  Can’t Tell |
| *CONSIDER:*   - *Were the baseline characteristics of each study group (e.g. age, sex, socio-economic group) clearly set out?* - *Were there any differences between the study groups that could affect the outcome/s?* | |
| 1. Apart from the experimental intervention, did each study group receive the same level of care (that is, were they treated equally)? | Yes  No  Can’t Tell |
| *CONSIDER:*   - *Was there a clearly defined study protocol?* - *If any additional interventions were given (e.g. tests or treatments), were they similar between the study groups?* - *Were the follow-up intervals the same for each study group?* | |
| **Section C: What are the results?** | |
| 1. Were the effects of intervention reported comprehensively? | Yes  No  Can’t Tell  **Frequency of AEs were summarized by preferred term and organ class term for each clinical sign using VeDDRA coding.** |
| *CONSIDER:*   - *Was a power calculation undertaken?* - *What outcomes were measured, and were they clearly specified?* - *How were the results expressed? For binary outcomes, were relative and absolute effects reported?* - *Were the results reported for each outcome in each study group at each follow-up interval?* - *Was there any missing or incomplete data?* - *Was there differential drop-out between the study groups that could affect the results?* - *Were potential sources of bias identified?* - *Which statistical tests were used?* - *Were p values reported?* | |
| 1. Was the precision of the estimate of the intervention or treatment effect reported? | Yes  No  Can’t Tell  **No statistical test was done for the safety assessment section of the study.** |
| *CONSIDER:*   - *Were confidence intervals (CIs) reported?* | |
| 1. Do the benefits of the experimental intervention outweigh the harms and costs? | Yes  No  Can’t Tell |
| *CONSIDER:*   - *What was the size of the intervention or treatment effect?* - *Were harms or unintended effects reported for each study group?* - *Was a cost-effectiveness analysis undertaken? (Cost-effectiveness analysis allows a comparison to be made between different interventions used in the care of the same condition or problem.)* | |
| **Section D: Will the results help locally?** | |
| 1. Can the results be applied to your local population/in your context? | Yes  No  Can’t Tell |
| *CONSIDER:*   - *Are the study participants similar to the people in your care?* - *Would any differences between your population and the study participants alter the outcomes reported in the study?* - *Are the outcomes important to your population?* - *Are there any outcomes you would have wanted information on that have not been studied or reported?* - *Are there any limitations of the study that would affect your decision?* | |
| 1. Would the experimental intervention provide greater value to the people in your care than any of the existing interventions? | Yes  No  Can’t Tell |
| *CONSIDER:*   - *What resources are needed to introduce this intervention taking into account time, finances, and skills development or training needs?* - *Are you able to disinvest resources in one or more existing interventions in order to be able to re-invest in the new intervention?* | |

| **APPRAISAL SUMMARY**: *List key points from your critical appraisal that need to be considered when assessing the validity of the results and their usefulness in decision-making.* | | |
| --- | --- | --- |
| **Positive/Methodologically sound** | **Negative/Relatively poor methodology** | **Unknowns** |
|  |  | **No radiograph taken post-treatment. Therefore, no records of the progression of OA.** |

**Supporting Method S3.**

CASP Checklist:

For Randomised Controlled Trials (RCTs)

| **Reviewer Name:** | Xingyu Yang |
| --- | --- |
| **Paper Title:** | A randomised, parallel-group clinical trial comparing bedinvetmab to meloxicam for the management of canine osteoarthritis |
| **Author:** | John F. Innes, B. Duncan X. Lascelles, Daniel Bell, Robert Tulloch, Alex McVey, Chad Northcott, Mahala Welbourn, Kate Higgins, Veronika Horakova, Thomas W. Maddox |
| **Web Link:** | <https://www.frontiersin.org/journals/veterinary-science/articles/10.3389/fvets.2025.1502218/full> |
| **Appraisal Date:** | 14/08/2025 |

| **Section A Is the basic study design valid for a randomised controlled trial?** | |
| --- | --- |
| 1. Did the study address a clearly formulated research question? | Yes  No  Can’t Tell |
| *CONSIDER:*  *Was the study designed to assess the outcomes of an intervention?*  *Is the research question ‘formulated’ in terms of:*   - *Population studied* - *Intervention given* - *Comparator chosen* - *Outcomes measured?* | |
| 1. Was the assignment of participants to interventions randomised? | Yes  No  Can’t Tell |
| *CONSIDER:*   - *How was randomisation carried out? Was the method appropriate?* - *Was randomisation sufficient to eliminate systematic bias?* - *Was the allocation sequence concealed from investigators and participants?* | |
| 1. Were all participants who entered the study accounted for at its conclusion? | Yes  No  Can’t Tell  **Two dogs were mistakenly included in randomisation as their initial visit COI was less than 26; these dogs (one in meloxicam group and one in bedinvetmab group) wee not included in safety analysis.** |
| *CONSIDER:*   - *Were losses to follow-up and exclusions after randomisation accounted for?* - *Were participants analysed in the study groups to which they were randomised (intention-to-treat analysis)?* - *Was the study stopped early? If so, what was the reason?* | |
| **Section B Was the study methodologically sound?** | |
| 1. (a) Were the participants ‘blind’ to intervention they were given? | Yes  No  Can’t Tell |
| (b) Were the investigators ‘blind’ to the intervention they were giving to participants? | Yes  No  Can’t Tell |
| (c) Were the people assessing/analysing outcome/s ‘blinded’? | Yes  No  Can’t Tell |
| 1. Were the study groups similar at the start of the randomised controlled trial? | Yes  No  Can’t Tell |
| *CONSIDER:*   - *Were the baseline characteristics of each study group (e.g. age, sex, socio-economic group) clearly set out?* - *Were there any differences between the study groups that could affect the outcome/s?* | |
| 1. Apart from the experimental intervention, did each study group receive the same level of care (that is, were they treated equally)? | Yes  No  Can’t Tell |
| *CONSIDER:*   - *Was there a clearly defined study protocol?* - *If any additional interventions were given (e.g. tests or treatments), were they similar between the study groups?* - *Were the follow-up intervals the same for each study group?* | |
| **Section C: What are the results?** | |
| 1. Were the effects of intervention reported comprehensively? | Yes  No  Can’t Tell |
| *CONSIDER:*   - *Was a power calculation undertaken?* - *What outcomes were measured, and were they clearly specified?* - *How were the results expressed? For binary outcomes, were relative and absolute effects reported?* - *Were the results reported for each outcome in each study group at each follow-up interval?* - *Was there any missing or incomplete data?* - *Was there differential drop-out between the study groups that could affect the results?* - *Were potential sources of bias identified?* - *Which statistical tests were used?* - *Were p values reported?* | |
| 1. Was the precision of the estimate of the intervention or treatment effect reported? | Yes  No  Can’t Tell |
| *CONSIDER:*   - *Were confidence intervals (CIs) reported?* | |
| 1. Do the benefits of the experimental intervention outweigh the harms and costs? | Yes  No  Can’t Tell |
| *CONSIDER:*   - *What was the size of the intervention or treatment effect?* - *Were harms or unintended effects reported for each study group?* - *Was a cost-effectiveness analysis undertaken? (Cost-effectiveness analysis allows a comparison to be made between different interventions used in the care of the same condition or problem.)* | |
| **Section D: Will the results help locally?** | |
| 1. Can the results be applied to your local population/in your context? | Yes  No  Can’t Tell |
| *CONSIDER:*   - *Are the study participants similar to the people in your care?* - *Would any differences between your population and the study participants alter the outcomes reported in the study?* - *Are the outcomes important to your population?* - *Are there any outcomes you would have wanted information on that have not been studied or reported?* - *Are there any limitations of the study that would affect your decision?* | |
| 1. Would the experimental intervention provide greater value to the people in your care than any of the existing interventions? | Yes  No  Can’t Tell |
| *CONSIDER:*   - *What resources are needed to introduce this intervention taking into account time, finances, and skills development or training needs?* - *Are you able to disinvest resources in one or more existing interventions in order to be able to re-invest in the new intervention?* | |

| **APPRAISAL SUMMARY**: *List key points from your critical appraisal that need to be considered when assessing the validity of the results and their usefulness in decision-making.* | | |
| --- | --- | --- |
| **Positive/Methodologically sound** | **Negative/Relatively poor methodology** | **Unknowns** |
| - **The study is the only published study that has compared bedinvetmab to an NSAID.** | - **The compliance for bedinvetmab was good because the injection was administered by vets during visits. However, the compliance for meloxicam was challenging to assess because the daily tablets were administered by owners themselves. The study intended to compare meloxicam to bedinvetmab, so the difference between administration method and frequency was inevitable. However, this does not dismiss the compliance issue encountered in the meloxicam group, which may have impacted the study result.** - **The most significant problem with the study methodology was the lack of blinding to both participants and outcome assessors. As stated above, the inherent difference between the two treatments made it difficult to blind the study, however, this was still an obvious confounding bias that could have impacted the study outcome. In addition, the investigators had already hypothesized bedinvetmab to be safer than meloxicam prior to the commencement of the study. Without blinding and with an expected outcome already in mind, the investigators may have introduced ascertainment bias when conducting the study.** | - **The study was only conducted for a period of 56 days, which was short for management of a chronic disease such as OA. The long-term safety of meloxicam compared to bedinvetmab is still largely unknown.** |

**Supporting Method S4.**

CASP Checklist:

For case control studies

| **Reviewer Name:** | Xingyu Yang |
| --- | --- |
| **Paper Title:** | Musculoskeletal adverse events in dogs receiving bedinvetmab (Librela) |
| **Author:** | Mike Farrell, Felix W. A. Waibel, Ines Carrera, Giliola Spattini, Louise Clark, Robert J. Adams, Dirsko J. F. Von Pfeil, Ricardo J. R. De Sousa, Diego Bobis Villagra |
| **Web Link:** | <https://www.frontiersin.org/journals/veterinary-science/articles/10.3389/fvets.2025.1581490/full> |
| **Appraisal Date:** | 14/08/2025 |

| **Section A: Are the results of the study valid?** | | |
| --- | --- | --- |
| 1. Did the study address a clearly focused issue? | Yes  No  Can’t Tell  **Population studied: MSAER reports submitted to EudraVigilance database.**  **Focused issue: does Librela’s MSAER rate exceed that of six comparator drugs with the same indication? The disproportionality analysis concludes a higher MSAER rate for bedinvetmab, so the issue is addressed appropriately.** | |
| *CONSIDER:*  *An issue can be ‘focused’ In terms of*   - *the population studied* - *whether the study tried to detect a beneficial or harmful effect* - *the risk factors studied* | | |
| 1. Did the authors use an appropriate method to answer their question? | Yes  No  Can’t Tell | |
| *CONSIDER:*   - *is a case control study an appropriate way of answering the question under the circumstances* - *did it address the study question* | | |
| 1. Were the cases recruited in an acceptable way? | Yes  No  Can’t Tell  **Cases were recruited from EuraVigilance database. Any report that specified co-administration of Librela were excluded. Any report that was due to confounding neurological and/or systemic/neoplastic diagnoses was excluded. Cases were clearly defined.**  **A total of 878 reports were incorporated into the analysis, which was a sufficient number.** | |
| *CONSIDER:*  *We are looking for selection bias which might compromise validity of the findings*   - *are the cases defined precisely* - *were the cases representative of a defined population (geographically and/or temporally)* - *was there an established reliable system for selecting all the cases* - *are they incident or prevalent* - *is there something special about the cases* - *is the time frame of the study relevant to disease/exposure* - *was there a sufficient number of cases selected* - *was there a power calculation* | | |
| 1. Were the controls selected in an acceptable way? | Yes  No  Can’t Tell  **A disproportionality analysis is a tool used in pharmacovigilance to identify potential safety signals. It is not exactly a case-control study, as there is no control group.** | |
| *CONSIDER:*  *We are looking for selection bias which might compromise the generalisability of the findings*   - *were the controls representative of the defined population (geographically and/or temporally)* - *was there something special about the controls* - *was the non-response high, could non-respondents be different in any way* - *are they matched, population based or randomly selected* - *was there a sufficient number of controls selected* | | |
| 1. Was the exposure accurately measured to minimise bias? | **Yes  No  Can’t Tell**  **Exposure defined as bedinvetmab or six other comparator drugs. Co-administration was excluded.**  **MSAERs were submitted by vets and other healthcare professionals, which were inherently subjective. But the exclusion method used by the disproportionality analysis was objective.**  **The analysis assessed the number of MSAER reports associated with bedinvetmab use or six other comparator drugs. The measurement reflected the frequency of adverse event reports.**  **No blinding was incorporated.**  **The recruited reports were submitted at least 3 months after Librela’s Europoean release. The temporal relation is correct.** | |
| *CONSIDER:*  *We are looking for measurement, recall or classification bias*   - *was the exposure clearly defined and accurately measured* - *did the authors use subjective or objective measurements* - *do the measures truly reflect what they are supposed to measure (have they been validated)* - *were the measurement methods similar in the cases and controls* - *did the study incorporate blinding where feasible* - *is the temporal relation correct (does the exposure of interest precede the outcome)* | | |
| 1. a) Aside from the exposure, did the groups have similar characteristics? | Yes  No  Can’t Tell  **The characteristics of dogs who experienced an adverse event were not described.** | |
| *CONSIDER:*  *List the ones you think might be important, that the author may have missed*   - *genetic* - *environmental* - *socio-economic* | | |
| 1. b) Have the authors taken account of the potential confounding factors in the design and/or in their analysis? | Yes  No  Can’t Tell  **The authors took into account of the possibility that MSAER would surge after a new drug is released. They looked for similar surges in Onsior, Previcox, and Galliprant, and still found no spike of MSAER reports as significant as that of Librela.** | |
| *CONSIDER:*   - *restriction in design, and techniques e.g. modelling, stratified-, regression-, or sensitivity analysis to correct, control or adjust for confounding factors* | | |
| **Section B: What are the results?** | | |
| 1. Was the treatment effect large? | | Yes  No  Can’t Tell  **Musculoskeletal MSAER reports associated with Librela was ~9 times more frequent than the combined total of six other comparator drugs. The effect was significant.** |
| *CONSIDER:*   - *what are the bottom-line results* - *is the analysis appropriate to the design* - *how strong is the association between exposure and outcome (look at the odds ratio)* - *are the results adjusted for confounding, and might confounding still explain the association* - *has adjustment made a big difference to the OR* | | |
| 1. Was the estimate of the treatment effect precise? | | Yes  No  Can’t Tell  **The difference between the report rate of Librela and comparator drugs was significant (~9 times more frequently in Librela), but the P value and CI were not calculated. The study also has lots of inherent biases such as no control, no blinding, and no randomization that have made any conclusion of lower level of evidence.** |
| *CONSIDER:*   - *size of the p-value* - *size of the confidence intervals* - *have the authors considered all the important variables* - *how was the effect of subjects refusing to participate evaluated* | | |
| 1. Do you believe the results? | | Yes  No  Can’t Tell |
| *CONSIDER:*   - *big effect is hard to ignore!* - *can it be due to chance, bias, or confounding* - *are the design and methods of this study sufficiently flawed to make the results unreliable* - *consider Bradford Hills criteria (e.g. time sequence, does-response gradient, strength, biological plausibility)* | | |
| **Section C: Will the results help locally?** | | |
| 1. Can the results be applied to your patients/the population of interest? | | Yes  No  Can’t Tell |
| *CONSIDER:*   - *the subjects covered in the study could be sufficiently different from your population to cause concern* - *if your local setting is likely to differ much from that of the study* - *can you quantify the local benefits and harms* | | |
| 1. Do the results of this study fit with other available evidence? | Yes  No  Can’t Tell  **Other existing RCTs have all concluded Librela safe to use.** | |
| *CONSIDER:*   - *all the available evidence from RCT’s Systematic Reviews, Cohort Studies, and Case Control Studies as well, for consistency* | | |

| **APPRAISAL SUMMARY**: *List key points from your critical appraisal that need to be considered when assessing the validity of the results and their usefulness in decision-making.* | | |
| --- | --- | --- |
| **Positive/Methodologically sound** | **Negative/Relatively poor methodology** | **Unknowns** |
| - **The authors considered of the possibility that MSAER would surge after a new drug is released. To account for this bias, they looked for similar surges when Onsior, Previcox, and Galliprant were first relseased and still found no spike of MSAER reports as significant as that of Librela.** | - **The study produced lower-level evidence of outcome because it had no control, no blinding, and no randomization. It was not adjusted for confounding and was subject to reporting bias.** - **The study directly compared the number of reports instead of the incidence rate of MSAEs because it did not take into account the number of dogs who have received treatment. This methodology is inherently problematic because a popular drug used widely can have more reports than a drug used rarely, without necessarily being more dangerous.** - **The methodology of filtering MSAEs from the database was not documented and was done by a single author.** |  |
